# Simultaneous Analysis of 19 Marker Components for Quality Control of Oncheong-Eum Using HPLC–DAD

**DOI:** 10.3390/molecules27092992

**Published:** 2022-05-06

**Authors:** Chang-Seob Seo, Hyeun-Kyoo Shin

**Affiliations:** KM Science Research Division, Korea Institute of Oriental Medicine, Daejeon 34054, Korea; hkshin@kiom.re.kr

**Keywords:** Oncheong-eum, traditional herbal prescription, method development, method validation, high-performance liquid chromatography

## Abstract

Oncheong-eum (OCE) is a traditional herbal prescription made by combining Samul-tang and Hwangryunhaedok-tang. It is primarily used to treat gynecological disorders such as metrorrhagia and metrostaxis. In the present study, we focused on developing and validating a simultaneous assay for the quality control of OCE using 19 marker components (gallic acid, 5-(hydroxymethyl)furfural, chlorogenic acid, geniposide, coptisine chloride, jatrorrhizine chloride, paeoniflorin, berberine chloride, palmatine chloride, ferulic acid, nodakenin, benzoic acid, baicalin, benzoylpaeoniflorin, wogonoside, baicalein, wogonin, decursin, and decursinol angelate). This analysis was performed using high-performance liquid chromatography coupled with a diode array detector, and chromatographic separation of the 19 markers was carried out using a SunFire^TM^ C_18_ reversed-phase column and gradient elution conditions with two mobile phases (0.1% aqueous formic acid–0.1% formic acid in acetonitrile). The developed analytical method was validated through linearity, limits of detection and quantification, recovery, and precision. Under this assay, 19 markers in OCE samples were detected at not detected–9.62 mg/g. The analytical methods developed and validated in our research will have value as basic data for the quality control of related traditional herbal prescriptions as well as OCE.

## 1. Introduction

In general, traditional herbal prescriptions consist of two or more herbal medicines. They have been used for a long time in Asian countries, especially Korea, China, and Japan, because of the advantages of fewer side effects and multicomponent/multitarget compared with synthetic drugs or Western medicine [1,2,3]. However, despite their prolonged use, modern scientific validation of their biological activity and research on standardization to maintain the consistency of raw materials are still needed.

Oncheong-eum (OCE; “Wen-Qing-Yin” in Chinese and “Unsei-in” in Japanese), also called “Haedoksamultang”, is a traditional herbal prescription that combines Samul-tang and Hwangryunhaedok-tang and consists of eight herbal medicines: *Angelica gigas* Nakai, *Cnidium officinale* Makino, *Paeonia lactiflora* Pall., *Rehmannia glutinosa* (Gaertn.) DC., *Coptis chinensis* Franch., *Phellodendron chinensis* C.K.Schneid., *Scutellaria baicalensis* Georgi, and *Gardenia jasminoides* Ellis, in the same weight ratio [4]. Since OCE was first recorded in the Manbyeonghoichun of Gong Tingxian, a physician in the Ming Dynasty, it has also been transmitted in Dongeuibogam of Heo Jun, a famous medical book of the Joseon Dynasty [4,5]. This prescription has been used to stop abnormal uterine bleeding, remove fever, and treat stomach pain. It is also used for skin diseases and metabolic diseases [5,6].

Various biological activities of OCE have been reported, such as the inhibition of cell proliferation in the human hepatocarcinoma cell line [7,8], inhibition of melanogenesis and tyrosinase activity in the murine melanoma cell line, skin regeneration and wrinkle improvement in the human normal fibroblast cell line [9], and pruritus [10,11].

OCE is composed of eight herbal medicines and contains numerous constituents. The major components are phenylpropanoids (e.g., chlorogenic acid) and coumarins (e.g., nodakenin, decursin, and decursinol angelate) from *A. gigas* [12], phenylpropanoids (e.g., ferulic acid) from *C. officinale* [13], monoterpenoids (e.g., albiflorin and paeoniflorin) from *P. lactiflora* [14], miscellaneous (e.g., 5-(hydroxymethyl)furfural) from *R. glutinosa* [15], alkaloids (e.g., berberine chloride) from *C. japonica* and *P. chinensis* [16,17], flavonoids (e.g., baicalin, and wogonoside) from *S. baicalensis* [18], and iridoid glycosides (e.g., geniposide) from *G. jasminoides* [19].

Several analytical methods are practiced for constant quality control of traditional herbal prescriptions using high-performance capillary electrophoresis [20], gas chromatography with mass spectrometry (GC–MS) [21], ultra-performance liquid chromatography (UPLC) [22], high-performance liquid chromatography combined with a diode array detector (HPLC–DAD) [23], HPLC with charged aerosol detector [24], and liquid chromatography–tandem mass spectrometry (LC–MS) [25]. Up until now, among various analytical techniques, the analytical method using HPLC–DAD equipment is the most widely used and recommended for the analysis of herbal medicines or herbal medicine prescriptions because of its wide selection of mobile phases, convenience of use, accuracy, and reproducibility of results [6]. A previous study reported a simultaneous analysis of six indicator compounds (berberine, baicalin, ferulic acid, geniposide, hydroxymethylfurfural, and paeoniflorin) in Wen-Qing-Yin using HPLC–DAD [6].

Thus, in this study, a simultaneous determination method for continuous quality control of OCE by HPLC–DAD using 19 markers—gallic acid (**1**), 5-(hydroxymethyl)furfural (**2**), chlorogenic acid (**3**), geniposide (**4**), coptisine chloride (**5**), jatrorrhizine chloride (**6**), paeoniflorin (**7**), berberine chloride (**8**), palmatine chloride (**9**), ferulic acid (**10**), nodakenin (**11**), benzoic acid (**12**), baicalin (**13**), benzoylpaeoniflorin (**14**), wogonoside (**15**), baicalein (**16**), wogonin (**17**), decursin (**18**), and decursinol angelate (**19**)—selected from each raw herbal medicine constituting OCE was developed, and the assay was validated.

## 2. Results and Discussion

### 2.1. Identification of the Major Components of Each Herbal Medicine Constituting OCE

To develop a simultaneous analysis method for the quality control of OCE using HPLC–DAD, the primary ingredients contained in eight raw herbal medicines were first explored. The ingredients selected for analysis in each raw herb are as follows: chlorogenic acid, nodakenin, decursin, and decursinol angelate from *A. gigas*; ferulic acid from *C. officinale*; albiflorin, paeoniflorin, benzoic acid, gallic acid, and benzoylpaeoniflorin from *P. lactiflora*; 5-(hydroxymethyl)furfural from *R. glutinosa*; berberine chloride, coptisine chloride, jatrorrhizine chloride, and palmatine chloride from *C. japonica* and *P. chinensis*; baicalin, baicalein, wogonin, and wogonoside from *S. baicalensis*; and geniposide and gardenoside from *G. jasminoides*. As a result of a comparative analysis of each constituent herbal medicine and their main components in HPLC using a reversed-phased C_18_ column and a distilled water (DW)–acetonitrile (ACN) mobile system (both containing 0.1% formic acid; FA), it was confirmed that the target components were present in each constituent herbal medicine (Figure S1).

### 2.2. Selection of Marker Analytes for Quality Control of OCE Using an HPLC Sytem

After performing as in Section 3.1 and performing the same in the OCE sample, 19 compounds were detected, whereas two components, gardenoside, and albiflorin, were not detected (Figure S2A,B). Of these two components, gardenoside showed a peak at the same retention time as the standard compound, but as a result of UV spectrum comparison, it was identified as a different component and thus excluded from analysis (Figure S2C). In addition, albiflorin was detected in the sample, but separation from other neighboring components was not complete, so it was not selected as a marker component. Finally, among the 21 components, 19 compounds completely isolated and identified from the OCE sample were selected as marker components suitable for the quality control of OCE. The chemical structures of compounds **1**–**19** selected as marker analytes for the quality control in OCE are shown in Figure S3.

### 2.3. Optimization of HPLC Analysis Conditions

**Figure 1:**
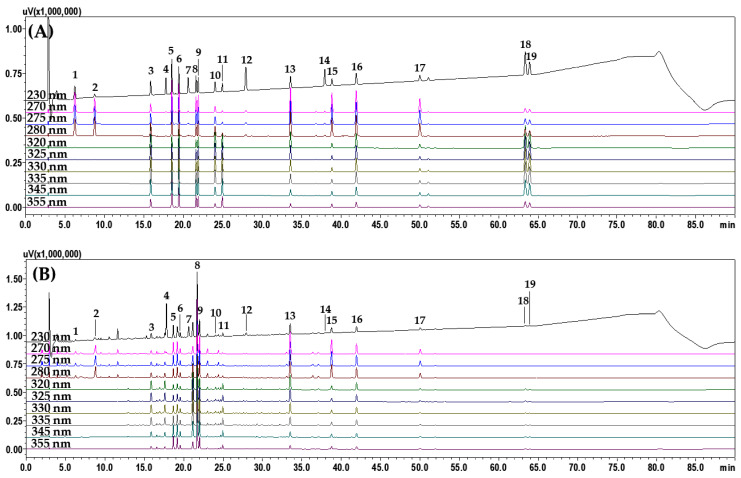
HPLC chromatograms of the standard solution (**A**) and OCE–1 sample (**B**). Gallic acid (**1**), 5-(hydroxymethyl)furfural (**2**), chlorogenic acid (**3**), geniposide (**4**), coptisine chloride (**5**), jatrorrhizine chloride (**6**), paeoniflorin (**7**), berberine chloride (**8**), palmatine chloride (**9**), ferulic acid (**10**), nodakenin (**11**), benzoic acid (**12**), baicalin (**13**), benzoylpaeoniflorin (**14**), wogonoside (**15**), baicalein (**16**), wogonin (**17**), decursin (**18**), and decursinol angelate (**19**). The concentration of each marker in the mixed standard solution is as follows: 10.00 μg/mL (compounds **2**, **8**–**10**, and **17**), 20.00 μg/mL (compounds **3**, **6**, **11**, **12**, **15**, and **16**), 30.00 μg/mL (compounds **1**, **4**, **5**, and **13**), 40.00 μg/mL (compounds **14**, **18**, and **19**), and 50.00 μg/mL (compound **7**).

We compared several conditions to search for efficient chromatographic separation conditions for the 19 marker analytes selected to achieve the development of an optimal HPLC simultaneous method for the quality control of OCE. First, the resolution of markers was compared by comparing reversed-phase C_18_ columns (4.6 mm × 250 mm, 5 μm) such as SunFire^TM^ (Waters, Milford, MA, USA), Gemini (Phenomenex, Torrance, CA, USA), Capcellpak UG80 (Shiseido, Tokyo, Japan), and Hypersil GOLD (Thermo Fisher Scientific Inc., San Jose, CA, USA). Next, the types of acid added to the mobile phase (FA, trifluoroacetic acid; TFA, acetic acid; AA, and phosphoric acid; PA), and the temperatures of the column oven (30, 35, 40, and 45 °C) were also compared. Figure 1 shows the representative HPLC chromatogram measured under the optimal analysis conditions finally selected (SunFire^TM^ C_18_ column, column temperature of 40 °C, and mobile phase system of 0.1% FA in DW–0.1% FA in ACN, based on the comparison and search conditions). All analytes were eluted within 65 min without the influence of neighboring peaks with a resolution of 16.9 or higher (Table S3).

### 2.4. Method Validation of the Developed HPLC Analytical Method

We developed an HPLC simultaneous analysis method using the 19 marker analytes for the efficient quality control of OCE. The developed assay was tested with several parameters, including linearity, the limit of detection (LOD), the limit of quantification (LOQ), and precision for validation. As shown in Table 1, the coefficient of determination (*r*^2^), which evaluates linearity, showed excellent linearity from 0.9999 to 1.0000 in all markers based on the prepared calibration curve. The LODs and LOQs of the 19 investigated marker components were 0.005–0.094 μg/mL and 0.015–0.285 μg/mL, respectively (Table 1). Recoveries (%) of compounds **1**–**19** ranged from 95.27% to 105.44% and are summarized in Table 2. The validation of precision was evaluated by the relative standard deviation (RSD (%)). As a result, all RSD values in the repeatability and intraday and interday precisions of investigated markers were ≤2.40%, showing suitable precision validation results (Table S3 and Table 3). These validation data indicate that the developed HPLC assay is an appropriate and accurate method for determining the marker substances selected for the quality control of OCE.

### 2.5. System Suitability and Stability Tests

The system suitability values were 1.17–21.22 (capacity factor; *k′*), 1.01–1.75 (selectivity factor; *α*), 18,808.16–1,737,667.55 (number of theoretical plates; *N*), 1.69–28.98 (resolution; *Rs*), and 1.01–1.18 (tailing factor; *Tf*). furthermore, the stability of each marker analyte measured using a standard solution for three days (0, 24, and 48 h) showed an RSD value in the range of 0.68% to 2.36%. The detailed results for each marker component are summarized in Table S4.

### 2.6. Quantification of the 19 Marker Compounds in OCE Samples

The developed HPLC analytical assay was successfully applied to simultaneous quantitation of the 19 markers in the OCE samples. Nineteen marker compounds were simultaneously monitored at 230 nm (compounds **4**, **7**, **12**, and **14**), 270 nm (compound **4**), 275 nm (compounds **13** and **15**–**17**), 280 nm (compound **2**), 320 nm (compound **10**), 325 nm (compound **3**), 330 nm (compounds **18** and **19**), 335 nm (compound **11**), 345 nm (compounds **6**, **8**, and **9**), and 355 nm (compound **5**). The amounts of the 19 investigated components in one water extract (OCE–1) and four commercial samples (OCE–2 to OCE–5, Figure S4) are presented in Table 4.

Table 4 shows that compounds **4** (1.61–9.62 mg/g), **7** (2.42–6.14 mg/g), **8** (1.59–7.21 mg/g), and **13** (4.43–8.59 mg/g) have higher concentrations than other investigated analytes in all samples. These four components are the main components of *G. jasminoides*, *P. lactiflora*, *C. japonica*, *P. chinensis*, and *S. baicalensis*.

## 3. Materials and Methods

### 3.1. Plant Materials

The eight raw herbal ingredients shown in Table S1 were purchased from Kwangmyungdang Pharmaceutical (Ulsan, Korea), a specialized herbal medicine manufacturing company, in November 2017. Each herbal medicine was used after morphological identification according to the guideline “The Dispensatory on the Visual and Organoleptic Examination of Herbal Medicine” by Dr. Goya Choi, Korea Institute of Oriental Medicine (KIOM, Daejeon, Korea) [26]. Each herbal medicine was deposited at the KM Science Research Division, KIOM (specimen no.: from 2018KE81–1 to 2018KE81–8).

### 3.2. Chemicals and Reagents

All reference standard compounds used for HPLC analysis were purchased from standard compound manufacturers: compounds **1** (CAS No. 149-91-7, 100.0%, Catalog No. G7384), **2** (CAS No. 67-47-0, ≥99.0%, Catalog No. W501808), **3** (CAS No. 327-97-9, 99.7%, Catalog No.PHL89175), and **12** (CAS No. 65-85-0, 99.9%, Catalog No. 242381) from KGaA (Darmstadt, Germany); compounds **4** (CAS No. 24512-63-8, ≥98.0%, Catalog No. 073-05891), **5** (CAS No. 6020-1804, ≥98.0%, Catalog No. 038-22001), **10** (CAS No. 1135-24-6, 98.0%, Catalog No. 086-04282), **13** (CAS No. 21967-41-9, 98.0%, Catalog No.024-15691), and **17** (CAS No. 632-85-9, ≥98.9%, Catalog No. 236-02321) from Fujifilm Wako Pure Chemical Co., Ltd. (Osaka, Japan); compounds **6** (CAS No. 6681-15-8, 98.4%, Catalog No. D91304201), **7** (CAS No. 23180-57-6, 99.4%, Catalog No. DR10579), **8** (CAS No. 633-65-8, 98.9%, Catalog No. DR10793), **14** (CAS No. 38642-49-8, ≥98.0%, Catalog No. DR10582), **15** (CAS No. 51059-44-0, 98.9%, Catalog No. DR10630), **16** (CAS No. 491-67-8, 99.4%, Catalog No. DR10625), and **18** (CAS No. 5928-25-6, 98.7%, Catalog No. DR11193) from Shanghai Sunny Biotech Co., Ltd. (Shanghai, China); compound **9** (CAS No. 10605-02-4, 99.3%, Catalog No. P2138) from Tokyo Chemical Industry Co., Ltd. (Tokyo, Japan); compound **11** (CAS No. 495-31-8, 99.5%, Catalog No. CFN90232) from ChemFaces Biochemical Co., Ltd. (Wuhan, China), and compound **19** (CAS No. 130848-06-5, 98.3%, Catalog No. BP1812) from Biopurify Phytochemicals (Chengdu, China). Methanol (MeOH), ACN, and DW (all HPLC grade) used for the preparation of test solutions, standard solutions, and chromatographic separation of marker analytes were purchased from JT Baker (Phillipsburg, NJ, USA). Acids, TFA (≥99.0%, HPLC grade) and AA (≥100.0%, ACS reagent grade) were purchased from Merck KGaA (Darmstadt, Germany), and FA (99.5%, HPLC grade) and PA (85.0%, ACS reagent grade) were purchased from Fujifilm Wako Pure Chemical Co., Ltd. (Osaka, Japan). All of these acids were used to add to the mobile phase. Dimethyl sulfoxide (DMSO, ≥99.9%, ACS reagent grade) was purchased from Merck KGaA (Darmstadt, Germany).

### 3.3. Preparation of OCE Water Extract

OCE water extract (OCE–1) was prepared at KIOM according to a previously reported manufacturing method [27,28]. That is, after mixing the same amount (each 625.0 g) of the eight herbal medicines shown in Table S1, 50 L of DW was added, and the mixture was extracted for 2 h at 100 °C using an electric extractor, COSMOS-660 (Kyungseo E&P, Incheon, Korea). According to the previously reported manufacturing method. Subsequently, the extracted water solution was filtered using a sieve (53 μm mesh). As a final step for producing a powder sample, the filtered extract was freeze-dried using an LP100R freeze dryer (IlShinBioBase, Yangju, Korea) (1232.6 g, yield 24.7%). Apart from the sample prepared by KIOM, the other four commercial samples (from OCE–2 to OCE–5) were purchased from different pharmaceutical companies, Kyungbang (Incheon, Korea), Jungwoo Medicines (Asan, Korea), Hankookshinyak (Nonsan, Korea), and Tsumura & Co. (Tokyo, Japan), respectively.

### 3.4. Preparation of Test Solutions and Standard Solutions for HPLC–DAD Analysis

For the test solutions for simultaneous determination of the 19 markers in OCE, about 100 mg was accurately taken of each prepared OCE water extract and commercially available products in a 10 mL volumetric flask, then filled with 70% MeOH (100 mg/10 mL). The continuously mixed samples were subjected to ultrasonic extraction at room temperature for 60 min. For the quantitative analysis of compounds **4**, **7**, **8**, and **13**, the prepared test solution was diluted 10-fold and used. All solutions were filtered before analysis using a 0.2 μm syringe filter (Pall Life Sciences, Ann Arbor, MI, USA) and then injected into the HPLC instrument.

Standard solutions of the 19 reference standard compounds were prepared at a concentration of 1.0 mg/mL using methanol or DMSO–MeOH solution (1:1), and then used while refrigerated.

### 3.5. HPLC Instrument and Analysis Conditions

The HPLC instrument used for simultaneous analysis of the 19 markers in OCE was a Prominence LC-20A modular system (Shimadzu Co., Tokyo, Japan) consisting of a quaternary pump (LC-20AT), DAD (SPD-M20A), autosampler (SIL-20A), and column oven (CTO-20A). The system is operated and controlled by LabSolution software (Version 5.53, SP3, Kyoto, Japan). Chromatographic separation for simultaneous analysis of marker analytes was performed using a reversed-phase column, SunFire^TM^ C_18_ column (4.6 mm × 250 mm, 5 μm, Waters, Milford, MA, USA), and gradient elution with two mobile phases (0.1% FA in DW–0.1% FA in ACN). Other detailed analysis conditions, including gradient elution conditions, are shown in Table S2.

### 3.6. Validation of the Developed HPLC Analytical Method

The analytical HPLC method developed to be applied to the quality control of OCE was validated by measuring and confirming various parameters such as linearity, LOD, LOQ, recovery, and precision [29]. First, the linearity of each marker component was checked in the following concentration ranges (0.31–20.00 μg/mL for compounds **1**, **6**, **10**, **12**–**14**, and **17**–**19**; 0.47–30.00 μg/mL for compounds **2**–**4**, **7**, **8**, and **11**; 0.78–50.00 μg/mL for compounds **5**, **9**, **15**, and **16**) and evaluated through the *r*^2^ of the prepared calibration curve.

Second, LOD and LOQ were calculated by the following equations, respectively. LOD = 3.3 × *σ*/*S* and LOD = 10 × *σ*/*S*, where *σ* and *S* represent the standard deviation of the *y*-intercept and the slope of the calibration curve in the regression equation of each marker measured three times, respectively.

Third, the recovery was validated by the standard addition method. Briefly, after accurately taking a 100 mg OCE powder sample in a 10 mL volumetric flask, three concentration levels (low, medium, and high) of each known marker compound were added. After that, the pretreatment process was the same as the preparation of the sample solution in Section 2.4. Extraction recovery (%) was calculated using following equation: Recovery (%) = found amount/spiked amount × 100.

Finally, the precision was evaluated as the relative standard deviation (RSD) of intraday and interday precisions and repeatability. The intraday and interday precisions were evaluated by calculating the RSD after measuring five times each for one day and three consecutive days using a standard solution of three concentrations in which the 19 markers were mixed. Repeatability was evaluated by obtaining the RSD of each marker analyte’s retention time and peak area after six repeated measurements.

### 3.7. System Suitability and Stability Tests

The system suitability test was validated by evaluating the *k′*, *α*, *Rs*, *N*, and *Tf* to evaluate the normal operation of the analysis system [27]. Furthermore, the stability of each marker component was tested for three days (0, 24, and 48 h) at 21 ± 1 °C using a standard solution.

### 3.8. Statistical Analysis

Data were expressed as mean, SD, and RSD (%), using Microsoft Excel 2019 software (Microsoft Co., Redmond, WA, USA).

## 4. Conclusions

In this study, a simultaneous analysis method using convenient, accurate, and reproducible HPLC–DAD for the quality control of OCE was developed and validated. This analytical method has been successfully applied to qualitative and quantitative analysis for the quality control of OCE. These results can provide reference data for improving the quality standards of OCE and related traditional herbal prescriptions.

## Figures and Tables

**Table 1:** Detection wavelength, linear range, regression equation, *r*^2^, LOD, LOQ, and retention time for simultaneous analysis of each marker component (*n* = 3).

Analyte	Detection Wavelength (nm)	Linear Range (μg/mL)	Regression Equation ^a^ *y* = a*x* + b	*r* ^2^	LOD (μg/mL)	LOQ (μg/mL)	Retention Time (min)
**1**	270	0.31–20.00	*y* = 41,926.60*x* + 2559.71	0.9999	0.034	0.102	6.24
**2**	280	0.47–30.00	*y* = 91,675.22*x* + 4694.69	0.9999	0.046	0.141	8.76
**3**	325	0.47–30.00	*y* = 52,471.53*x* + 5007.02	0.9999	0.075	0.228	15.84
**4**	230	0.47–30.00	*y* = 21,695.37*x* + 3442.20	0.9998	0.076	0.230	17.79
**5**	355	0.78–50.00	*y* = 39,690.73*x* + 6737.30	0.9999	0.083	0.252	18.61
**6**	345	0.31–20.00	*y* = 53,377.60*x* + 2860.29	1.0000	0.073	0.220	19.49
**7**	230	0.47–30.00	*y* = 15,685.87*x* − 1318.46	0.9999	0.006	0.018	20.61
**8**	345	0.47–30.00	*y* = 51,279.59*x* + 5895.33	0.9999	0.020	0.061	21.67
**9**	345	0.78–50.00	*y* = 53,582.09*x* + 9136.34	0.9999	0.094	0.285	21.95
**10**	320	0.31–20.00	*y* = 84,394.31*x* + 7794.43	0.9999	0.026	0.078	24.03
**11**	335	0.47–30.00	*y* = 38,849.24*x* + 5106.67	0.9999	0.045	0.138	24.95
**12**	230	0.31–20.00	*y* = 70,000.98*x* + 4949.39	0.9999	0.005	0.015	27.93
**13**	275	0.31–20.00	*y* = 42,108.54*x* + 3779.66	0.9999	0.078	0.236	33.55
**14**	230	0.31–20.00	*y* = 21,287.06*x* + 626.36	1.0000	0.052	0.156	37.94
**15**	275	0.78–50.00	*y* = 56,245.50*x* + 12,765.82	0.9999	0.079	0.239	38.81
**16**	275	0.78–50.00	*y* = 64,528.60*x* + 6020.97	0.9999	0.086	0.262	41.93
**17**	275	0.31–20.00	*y* = 91,000.29*x* + 7853.01	0.9999	0.029	0.089	50.01
**18**	330	0.31–20.00	*y* = 41,162.22*x* + 2663.76	0.9999	0.015	0.045	63.38
**19**	330	0.31–20.00	*y* = 32,163.04*x* + 2483.09	0.9999	0.034	0.102	63.94

^a^*y*: peak area of compounds; *x*: concentration (μg/mL) of compounds. Gallic acid (**1**), 5-(hydroxymethyl)furfural (**2**), chlorogenic acid (**3**), geniposide (**4**), coptisine chloride (**5**), jatrorrhizine chloride (**6**), paeoniflorin (**7**), berberine chloride (**8**), palmatine chloride (**9**), ferulic acid (**10**), nodakenin (**11**), benzoic acid (**12**), baicalin (**13**), benzoylpaeoniflorin (**14**), wogonoside (**15**), baicalein (**16**), wogonin (**17**), decursin (**18**), and decursinol angelate (**19**).

**Table 2:** Recovery (%) of the 19 marker components in the developed HPLC method (*n* = 5).

Analyte	Spiked Amount (μg/mL)	Found Amount (μg/mL)	Recovery (%)	RSD (%)
**1**	1.00	1.01	101.07	1.80
	2.00	2.01	100.62	1.29
	4.00	4.12	102.89	1.14
**2**	2.00	2.05	102.36	0.66
	5.00	5.15	103.08	0.33
	10.00	10.34	103.36	0.29
**3**	2.00	2.01	100.66	0.73
	5.00	5.11	102.28	0.66
	10.00	10.35	103.49	0.48
**4**	2.00	1.97	98.32	1.32
	5.00	4.95	98.99	1.26
	10.00	10.28	102.81	0.81
**5**	3.00	3.05	101.80	0.83
	7.50	7.15	95.27	0.15
	15.00	14.63	97.56	0.42
**6**	1.00	1.00	99.78	0.60
	2.00	1.97	98.66	1.31
	4.00	3.95	98.65	1.69
**7**	2.00	1.97	98.74	0.61
	4.00	3.96	98.98	0.65
	8.00	8.25	103.16	0.54
**8**	2.00	2.00	99.93	1.36
	4.00	3.96	99.09	0.71
	8.00	7.86	98.29	0.40
**9**	4.00	4.07	101.76	1.70
	10.00	9.94	99.35	1.30
	20.00	20.18	100.90	0.24
**10**	1.00	1.02	102.18	1.10
	2.00	2.04	101.84	0.30
	4.00	4.14	103.54	0.34
**11**	2.00	2.06	103.05	2.21
	5.00	5.05	100.98	0.47
	10.00	10.39	103.88	0.58
**12**	1.00	1.03	102.74	2.37
	2.00	2.01	100.65	0.82
	4.00	3.87	96.87	0.50
**13**	1.00	0.98	97.81	0.59
	2.00	1.95	97.49	0.88
	4.00	3.88	96.93	0.69
**14**	1.00	1.02	102.10	1.09
	2.00	2.01	100.65	1.06
	4.00	4.09	102.36	0.64
**15**	4.00	4.22	105.44	1.09
	10.00	9.67	96.72	1.80
	20.00	20.07	100.37	0.37
**16**	3.00	2.97	98.99	0.64
	7.50	7.46	99.50	0.51
	15.00	14.99	99.94	0.29
**17**	1.00	0.98	98.33	1.01
	2.00	1.92	95.98	0.82
	4.00	3.98	99.58	0.70
**18**	1.00	1.03	102.59	0.87
	2.00	1.95	97.32	0.37
	4.00	4.14	103.54	0.17
**19**	1.00	1.04	103.67	0.77
	2.00	1.96	98.19	0.53
	4.00	4.08	101.93	0.24

Gallic acid (**1**), 5-(hydroxymethyl)furfural (**2**), chlorogenic acid (**3**), geniposide (**4**), coptisine chloride (**5**), jatrorrhizine chloride (**6**), paeoniflorin (**7**), berberine chloride (**8**), palmatine chloride (**9**), ferulic acid (**10**), nodakenin (**11**), benzoic acid (**12**), baicalin (**13**), benzoylpaeoniflorin (**14**), wogonoside (**15**), baicalein (**16**), wogonin (**17**), decursin (**18**), and decursinol angelate (**19**).

**Table 3:** Precision test of marker compounds **1**–**19** in the developed HPLC method.

Analyte	Conc. (μg/mL)	Intraday (*n* = 5)			Interday (*n* = 5)		
		Observed Conc. (μg/mL) ± SD	Precision (RSD, %)	Accuracy (%)	Observed Conc. (μg/mL) ± SD	Precision (RSD, %)	Accuracy (%)
**1**	5.0	5.05 ± 0.04	0.72	101.04	5.03 ± 0.10	2.09	100.52
	10.0	10.12 ± 0.11	1.06	101.24	10.15 ± 0.20	2.00	101.49
	20.0	19.85 ± 0.1	0.74	99.26	19.91 ± 0.31	1.56	99.56
**2**	7.5	7.62 ± 0.02	0.32	101.61	7.56 ± 0.05	0.72	100.82
	15.0	14.99 ± 0.06	0.38	99.93	15.05 ± 0.17	1.14	100.34
	30.0	30.19 ± 0.08	0.28	100.62	30.00 ± 0.20	0.66	100.02
**3**	7.5	7.51 ± 0.04	0.56	100.18	7.45 ± 0.18	2.40	99.36
	15.0	15.13 ± 0.10	0.69	100.86	15.08 ± 0.32	2.11	100.53
	30.0	29.66 ± 0.23	0.79	98.87	29.57 ± 0.55	1.85	98.55
**4**	7.5	7.65 ± 0.03	0.41	101.97	7.68 ± 0.06	0.76	102.44
	15.0	15.25 ± 0.03	0.20	101.65	15.48 ± 0.18	1.18	103.18
	30.0	30.04 ± 0.18	0.61	100.13	30.31 ± 0.58	1.91	101.03
**5**	12.5	12.83 ± 0.12	0.94	102.68	12.92 ± 0.23	1.80	103.39
	25.0	25.68 ± 0.23	0.88	102.70	26.04 ± 0.51	1.96	104.17
	50.0	50.26 ± 0.47	0.93	100.53	50.68 ± 1.01	2.00	101.36
**6**	5.0	5.08 ± 0.04	0.75	101.68	5.14 ± 0.09	1.77	102.74
	10.0	10.19 ± 0.05	0.52	101.89	10.36 ± 0.20	1.91	103.55
	20.0	20.05 ± 0.18	0.92	100.25	20.21 ± 0.38	1.89	101.04
**7**	7.5	7.53 ± 0.06	0.77	100.39	7.56 ± 0.07	0.92	100.77
	15.0	15.13 ± 0.10	0.65	100.86	15.17 ± 0.26	1.70	101.14
	30.0	30.01 ± 0.16	0.53	100.03	30.31 ± 0.46	1.51	101.05
**8**	7.5	7.71 ± 0.06	0.84	102.82	7.77 ± 0.13	1.73	103.57
	15.0	15.41 ± 0.15	0.99	102.75	15.64 ± 0.31	1.96	104.27
	30.0	30.14 ± 0.26	0.86	100.46	30.40 ± 0.60	1.97	101.34
**9**	12.5	12.80 ± 0.11	0.82	102.41	12.91 ± 0.23	1.75	103.24
	25.0	25.61 ± 0.18	0.72	102.43	26.00 ± 0.50	1.91	104.00
	50.0	49.98 ± 0.44	0.88	99.96	50.55 ± 1.03	2.04	101.10
**10**	5.0	5.13 ± 0.02	0.45	102.67	5.17 ± 0.09	1.71	103.44
	10.0	10.28 ± 0.10	0.96	102.81	10.39 ± 0.21	2.01	103.93
	20.0	20.08 ± 0.19	0.94	100.42	20.21 ± 0.39	1.95	101.06
**11**	7.5	7.71 ± 0.04	0.49	102.74	7.78 ± 0.14	1.75	103.74
	15.0	15.43 ± 0.11	0.71	102.87	15.65 ± 0.29	1.88	104.36
	30.0	30.17 ± 0.25	0.84	100.57	30.41 ± 0.59	1.93	101.37
**12**	5.0	5.12 ± 0.02	0.48	102.45	5.11 ± 0.06	1.17	102.21
	10.0	10.18 ± 0.01	0.12	101.76	10.28 ± 0.10	0.98	102.79
	20.0	20.08 ± 0.13	0.65	100.41	20.14 ± 0.38	1.89	100.68
**13**	5.0	5.13 ± 0.04	0.82	102.69	5.17 ± 0.09	1.71	103.38
	10.0	10.31 ± 0.12	1.13	103.11	10.43 ± 0.19	1.84	104.26
	20.0	20.08 ± 0.22	1.11	100.41	20.21 ± 0.39	1.94	101.03
**14**	5.0	5.04 ± 0.03	0.54	100.74	5.08 ± 0.05	1.08	101.54
	10.0	9.91 ± 0.05	0.54	99.06	10.21 ± 0.23	2.28	102.06
	20.0	19.86 ± 0.10	0.49	99.28	20.11 ± 0.26	1.31	100.57
**15**	12.5	12.88 ± 0.11	0.88	103.05	12.97 ± 0.23	1.74	103.78
	25.0	25.81 ± 0.30	1.17	103.22	26.11 ± 0.48	1.85	104.45
	50.0	50.32 ± 0.54	1.08	100.63	50.64 ± 0.97	1.92	101.28
**16**	12.5	12.67 ± 0.11	0.86	101.36	12.76 ± 0.24	1.85	102.06
	25.0	25.42 ± 0.31	1.22	101.68	25.62 ± 0.48	1.86	102.47
	50.0	49.77 ± 0.45	0.90	99.54	49.87 ± 0.98	1.97	99.73
**17**	5.0	5.15 ± 0.04	0.84	102.99	5.18 ± 0.09	1.72	103.70
	10.0	10.32 ± 0.12	1.19	103.20	10.44 ± 0.19	1.85	104.38
	20.0	20.11 ± 0.21	1.05	100.53	20.24 ± 0.40	1.95	101.19
**18**	5.0	5.10 ± 0.05	0.89	102.08	5.14 ± 0.09	1.79	102.77
	10.0	10.28 ± 0.11	1.11	102.76	10.39 ± 0.19	1.85	103.93
	20.0	20.13 ± 0.19	0.96	100.63	20.24 ± 0.40	1.97	101.19
**19**	5.0	5.13 ± 0.04	0.84	102.58	5.17 ± 0.09	1.67	103.31
	10.0	10.31 ± 0.13	1.22	103.12	10.41 ± 0.19	1.83	104.15
	20.0	20.11 ± 0.20	0.98	100.55	20.22 ± 0.40	1.96	101.08

Gallic acid (**1**), 5-(hydroxymethyl)furfural (**2**), chlorogenic acid (**3**), geniposide (**4**), coptisine chloride (**5**), jatrorrhizine chloride (**6**), paeoniflorin (**7**), berberine chloride (**8**), palmatine chloride (**9**), ferulic acid (**10**), nodakenin (**11**), benzoic acid (**12**), baicalin (**13**), benzoylpaeoniflorin (**14**), wogonoside (**15**), baicalein (**16**), wogonin (**17**), decursin (**18**), and decursinol angelate (**19**).

**Table 4:** Amounts of the 19 marker components in OCE samples (*n* = 3).

Analyte	OCE–1		OCE–2		OCE–3		OCE–4		OCE–5	
	Mean (mg/g)	RSD (%)	Mean (mg/g)	RSD (%)	Mean (mg/g)	RSD (%)	Mean (mg/g)	RSD (%)	Mean (mg/g)	RSD (%)
**1**	0.67	0.20	0.20	2.20	0.75	0.15	0.44	0.41	0.24	0.46
**2**	1.10	0.21	0.08	0.24	0.04	1.38	<LOQ	–	<LOQ	–
**3**	1.20	0.17	0.11	0.85	0.03	0.73	<LOQ	–	0.32	0.70
**4**	9.62	0.32	1.61	0.20	1.87	0.33	1.76	1.73	2.73	0.12
**5**	1.72	0.65	<LOQ	–	0.04	2.05	0.26	0.43	0.79	0.38
**6**	0.56	0.25	<LOQ	–	<LOQ	–	0.07	0.99	0.18	0.12
**7**	6.14	0.63	2.76	0.23	2.42	0.29	2.85	0.14	4.14	0.37
**8**	7.21	0.67	2.01	0.18	1.68	0.04	1.59	0.06	3.90	0.03
**9**	1.89	0.24	0.07	0.97	0.09	1.27	0.32	0.36	0.60	0.33
**10**	0.23	0.32	0.07	0.21	<LOQ	–	0.02	0.51	0.14	0.59
**11**	1.21	0.23	0.18	0.65	0.07	0.24	0.03	0.61	0.02	2.03
**12**	0.41	0.47	0.13	0.86	0.24	1.04	0.21	1.09	0.17	0.49
**13**	4.43	1.28	6.12	0.25	8.06	0.04	8.59	0.19	7.51	0.08
**14**	0.16	0.75	0.09	1.10	0.05	1.53	0.05	2.06	0.14	2.05
**15**	2.20	0.66	1.41	0.17	0.24	0.24	1.52	0.95	1.45	0.98
**16**	1.46	2.08	0.37	1.21	0.21	0.19	0.09	0.76	0.01	1.66
**17**	0.57	0.51	0.10	0.18	0.06	0.22	0.05	0.60	0.04	0.39
**18**	0.24	0.69	0.09	0.44	0.05	0.83	0.01	2.16	ND	–
**19**	0.19	0.57	0.10	0.63	0.06	1.01	0.02	1.68	ND	–

## Data Availability

All data can be found in this paper.

## Supplementary Materials

The following supporting information can be downloaded at: https://www.mdpi.com/article/10.3390/molecules27092992/s1. Figure S1: HPLC chromatogram of constituent herbal medicines and their main components; Figure S2: HPLC chromatograms of the solution of the standard mixture (A) and 70% methanol solution of OCE–1 sample (B), and UV spectrum of gardenoside (C); Figure S3: Chemical structures of the selected 19 marker compounds for the quality control of OCE; Figure S4: HPLC chromatograms of OCE–2 to OCE–5 samples; Table S1: Composition of OCE; Table S2: HPLC chromatographic conditions for analyzing the 19 markers of OCE; Table S3. Repeatability of compounds **1**–**19** (*n* = 6); Table S4: System suitability and stability of compounds **1**–**1****9**.

## Abbreviations

*α*	Selectivity factor
AA	Acetic acid
ACN	Acetonitrile
DMSO	Dimethyl sulfoxide
DW	Distilled water
FA	Formic acid
GC–MS	Gas chromatography with mass spectrometry
HPLC–DAD	High-performance liquid chromatography combined with a diode array detector
*k* *′*	Capacity factor
KIOM	Korea Institute of Oriental Medicine
LC-MS	Liquid chromatography–tandem mass spectrometry
LOD	Limit of detection
LOQ	Limit of quantification
MeOH	Methanol
*N*	Number of theoretical plates
OCE	Oncheong-eum
PA	Phosphoric acid
*r* ^2^	Coefficient of determination
*Rs*	Resolution
RSD	Relative standard deviation
*Tf*	Tailing factor
TFA	Trifluoroacetic acid
UPLC	Ultra-performance liquid chromatography
