# Supplementary material for: Simultaneous Analysis of 19 Marker Components for Quality Control of Oncheong-Eum Using HPLC–DAD
Source: Molecules. 2022 May 6;27(9):2992. doi: 10.3390/molecules27092992 (PMC9104317; doi:10.3390/molecules27092992)
Supplement: Supplementary file 1 [file molecules-27-02992-s001.zip › molecules-1719588-supplementary_Proofreading_220507.pdf]

Table S1. Composition of OCE.

English Name	Scientific Name	Family	Using Part	Origin	Amount (g)	Ratio (%)
Angelicae Gigas Root	*Angelica gigas* Nakai	Apiaecae	Root	Pyeongchang, Korea	625.0	12.5
Cnidium Rhizome	*Cnidium officinale* Makino	Umbelliferae	Rhizome	Yeongyang, Korea	625.0	12.5
Paoy Root	*Paeonia lactiflora* Pall.	Paeoniaceae	Root	Uiseong, Korea	625.0	12.5
Prepared Rehmannia Root	*Rehmannia glutinosa* (Gaertn.) DC.	Plantaginaceae	Root	Jeongeup, Korea	625.0	12.5
Coptis Rhizome	*Coptis chinensis* Franch.	Ranunculaceae	Rhizome	China	625.0	12.5
Phellodendron Bark	*Phellodendron chinensis* C.K.Schneid.	Rutaceae	Bark	China	625.0	12.5
Scutellaria Root	*Scutellaria baicalensis* Georgi	Lamiaceae	Root	Yeosu, Korea	625.0	12.5
Gardenia Fruit	*Gardenia jasminoides* Ellis	Rubiaceae	Fruit	Imsil, Korea	625.0	12.5
				Total	5000.0	100.0

Table S2. HPLC chromatographic conditions for analyzing the 19 markers of OCE.

**Chromatographic Parameter**			
Column	SunFire™ C_{18} analytical column (250 mm × 4.6 mm, 5 μm)		
Detector	DAD (230, 270, 275, 280, 320, 325, 330, 335, 345, and 355 nm)		
Flow rate (mL/min)	1.0		
Injection volume (μL)	10.0		
Column temperature (°C)	40.0		
Mobile phase	A: 0.1% (v/v) formic acid in DW<br>B: 0.1% (v/v) formic acid in ACN		
Gradient elution	Time (min)	A (%)	B (%)
	0	95	5
	60	40	60
	70	10	90
	75	10	90
	80	95	5
	90	95	5

Table S3. Repeatability of compounds **1–19** (n = 6).

Analyte	RSD (%) of retention time	RSD (%) of peak area
**1**	0.10	0.31
**2**	0.04	0.55
**3**	0.05	0.26
**4**	0.03	0.45
**5**	0.21	0.26
**6**	0.18	0.22
**7**	0.02	0.47
**8**	0.18	0.35
**9**	0.16	0.13
**10**	0.01	0.21
**11**	0.02	0.22
**12**	0.01	0.23
**13**	0.07	0.24
**14**	0.01	0.34
**15**	0.06	0.23
**16**	0.01	0.35
**17**	0.01	0.23
**18**	0.01	0.25
**19**	0.01	0.24

Gallic acid (**1**), 5-(hydroxymethyl)furfural (**2**), chlorogenic acid (**3**), geniposide (**4**), coptisine chloride (**5**), jatrorrhizine chloride (**6**), paeoniflorin (**7**), berberine chloride (**8**), palmatine chloride (**9**), ferulic acid (**10**), nodakenin (**11**), benzoic acid (**12**), baicalin (**13**), benzoylpaeoniflorin (**14**), wogonoside (**15**), baicalein (**16**), wogonin (**17**), decursin (**18**), and decursinol angelate (**19**).

Table S4. System suitability and stability of compounds **1–19**.

**Analyte**	*k′*	*α*	*N*	*Rs*	*Tf*	**Stability** [a]
**1**	1.17	1.75	18808.16	8.78	1.04	1.90
**2**	2.04	1.75	50406.01	8.78	1.04	0.68
**3**	4.51	1.15	288970.02	10.06	1.12	2.36
**4**	5.18	1.05	433890.57	4.24	1.07	0.87
**5**	5.44	1.06	528614.53	5.35	1.11	1.65
**6**	5.75	1.07	683541.95	6.42	1.13	1.72
**7**	6.16	1.06	449183.30	5.40	1.18	1.00
**8**	6.51	1.02	827601.97	1.77	1.10	1.54
**9**	6.61	1.02	905559.11	1.77	1.13	1.64
**10**	7.35	1.04	506901.81	4.39	1.05	1.53
**11**	7.67	1.04	807990.44	4.39	1.07	1.60
**12**	8.71	1.14	436944.47	12.58	1.03	0.94
**13**	10.68	1.14	948111.45	18.66	1.13	1.60
**14**	12.19	1.03	1227146.45	3.80	1.13	1.02
**15**	12.50	1.03	1148533.50	3.80	1.09	1.58
**16**	13.57	1.09	1155845.52	12.07	1.09	1.68
**17**	16.38	1.21	1336977.56	28.98	1.02	1.55
**18**	21.03	1.01	1707521.47	1.69	1.01	1.77
**19**	21.22	1.01	1737667.55	1.69	1.04	1.57

[a] Stability was expressed as RSD (%). Gallic acid (**1**), 5-(hydroxymethyl)furfural (**2**), chlorogenic acid (**3**), geniposide (**4**), coptisine chloride (**5**), jatrorrhizine chloride (**6**), paeoniflorin (**7**), berberine chloride (**8**), palmatine chloride (**9**), ferulic acid (**10**), nodakenin (**11**), benzoic acid (**12**), baicalin (**13**), benzoylpaeoniflorin (**14**), wogonoside (**15**), baicalein (**16**), wogonin (**17**), decursin (**18**), and decursinol angelate (**19**).

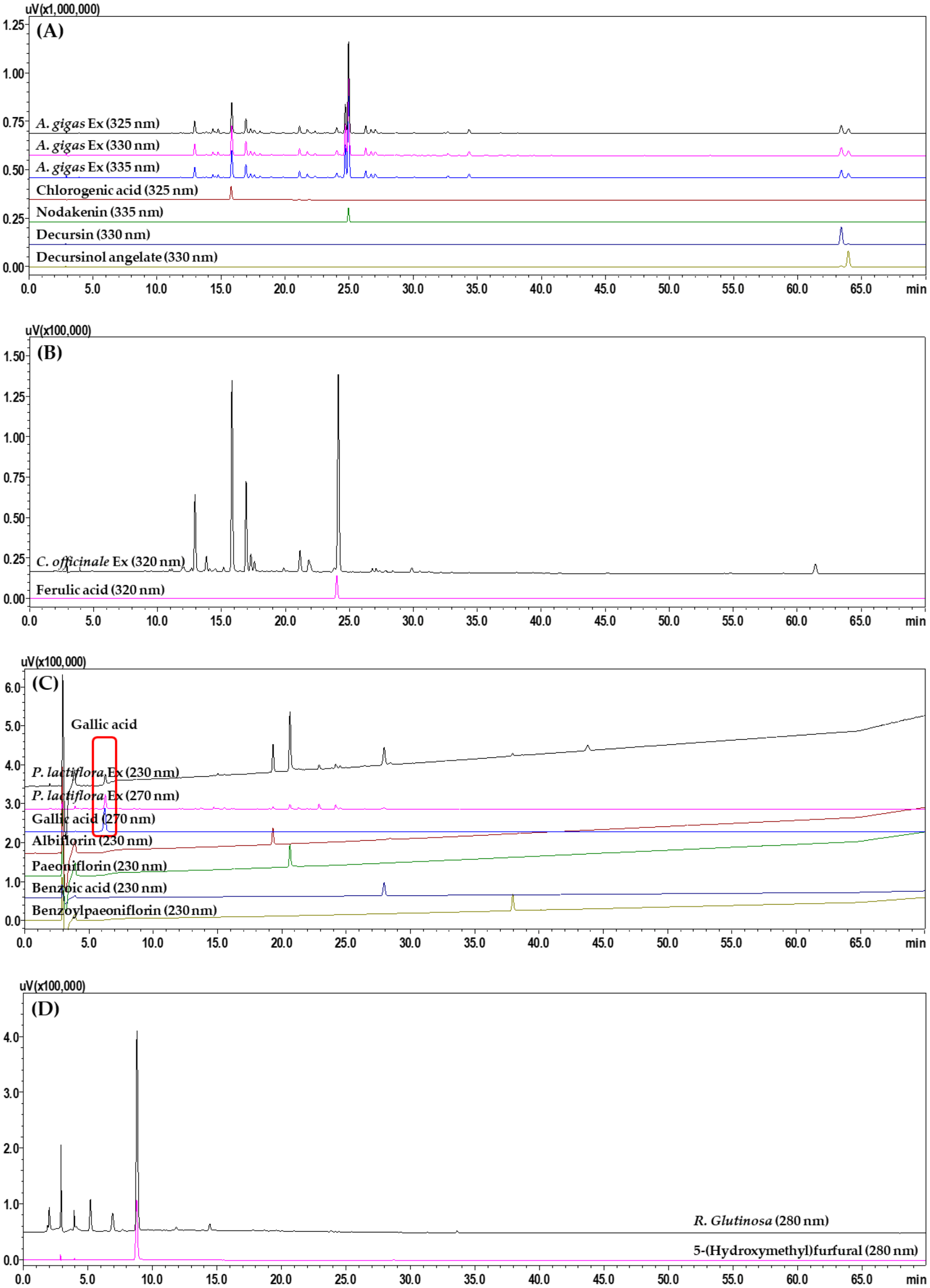
(A)
uV(x1,000,000)
A. gigas Ex (325 nm)
A. gigas Ex (330 nm)
A. gigas Ex (335 nm)
Chlorogenic acid (325 nm)
Nodakenin (335 nm)
Decursin (330 nm)
Decursinol angelate (330 nm)
min
(B)
uV(x100,000)
C. officinale Ex (320 nm)
Ferulic acid (320 nm)
min
(C)
uV(x100,000)
Gallic acid
P. lactiflora Ex (230 nm)
P. lactiflora Ex (270 nm)
Gallic acid (270 nm)
Albiflorin (230 nm)
Paeoniflorin (230 nm)
Benzoic acid (230 nm)
Benzoylpaeoniflorin (230 nm)
min
(D)
uV(x100,000)
R. Glutinosa (280 nm)
5-(Hydroxymethyl)furfural (280 nm)
min

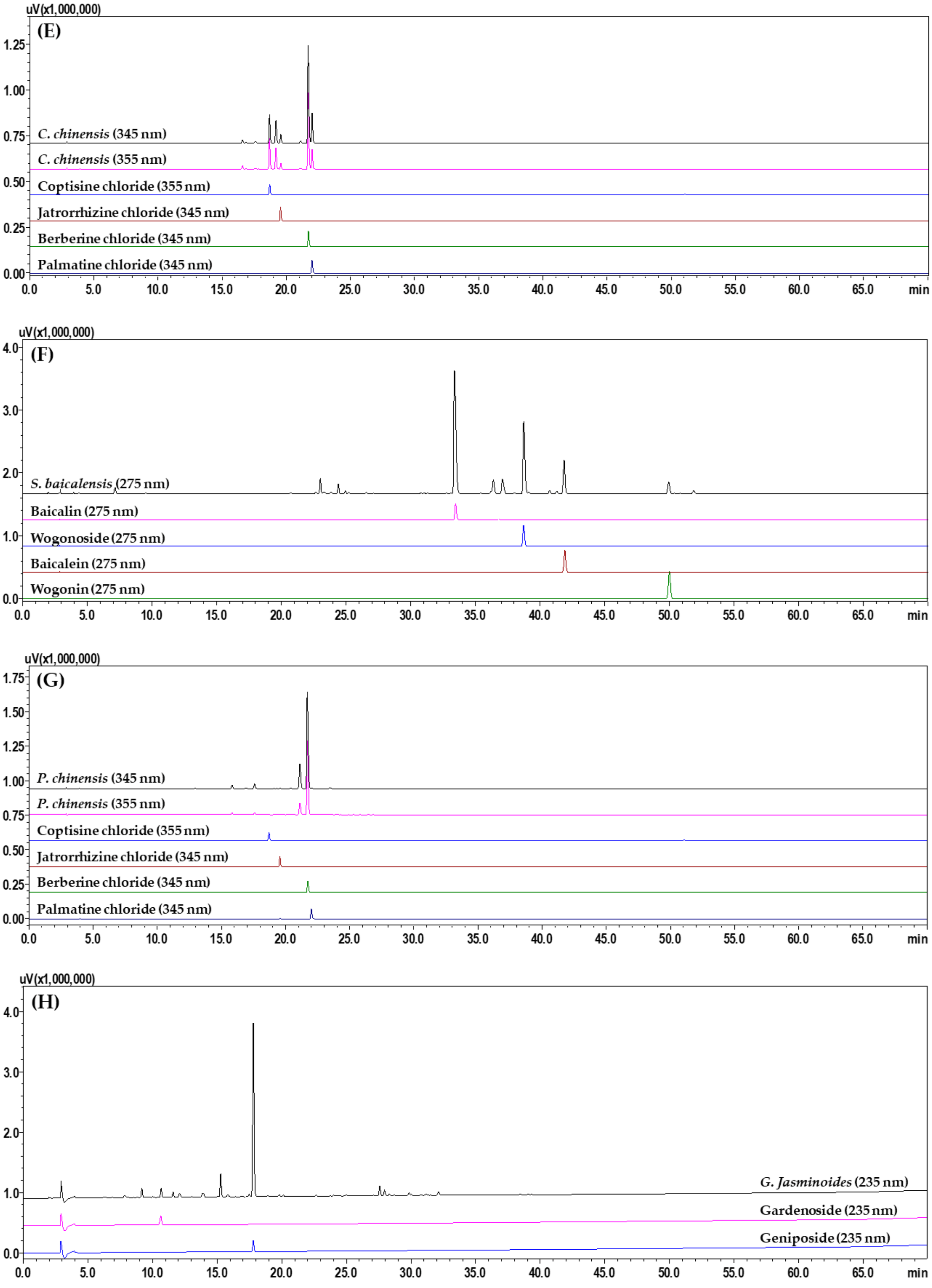

Figure S1. HPLC chromatogram of constituent herbal medicines and their main components. A: *A. gigas*, B: *C. officinale*, C: *P. lactiflora*, D: *R. glutinosa*, E: *C. chinensis*, F: *S. baicalensis*, G: *P. chinensis*, H: *G. jasminoides*.

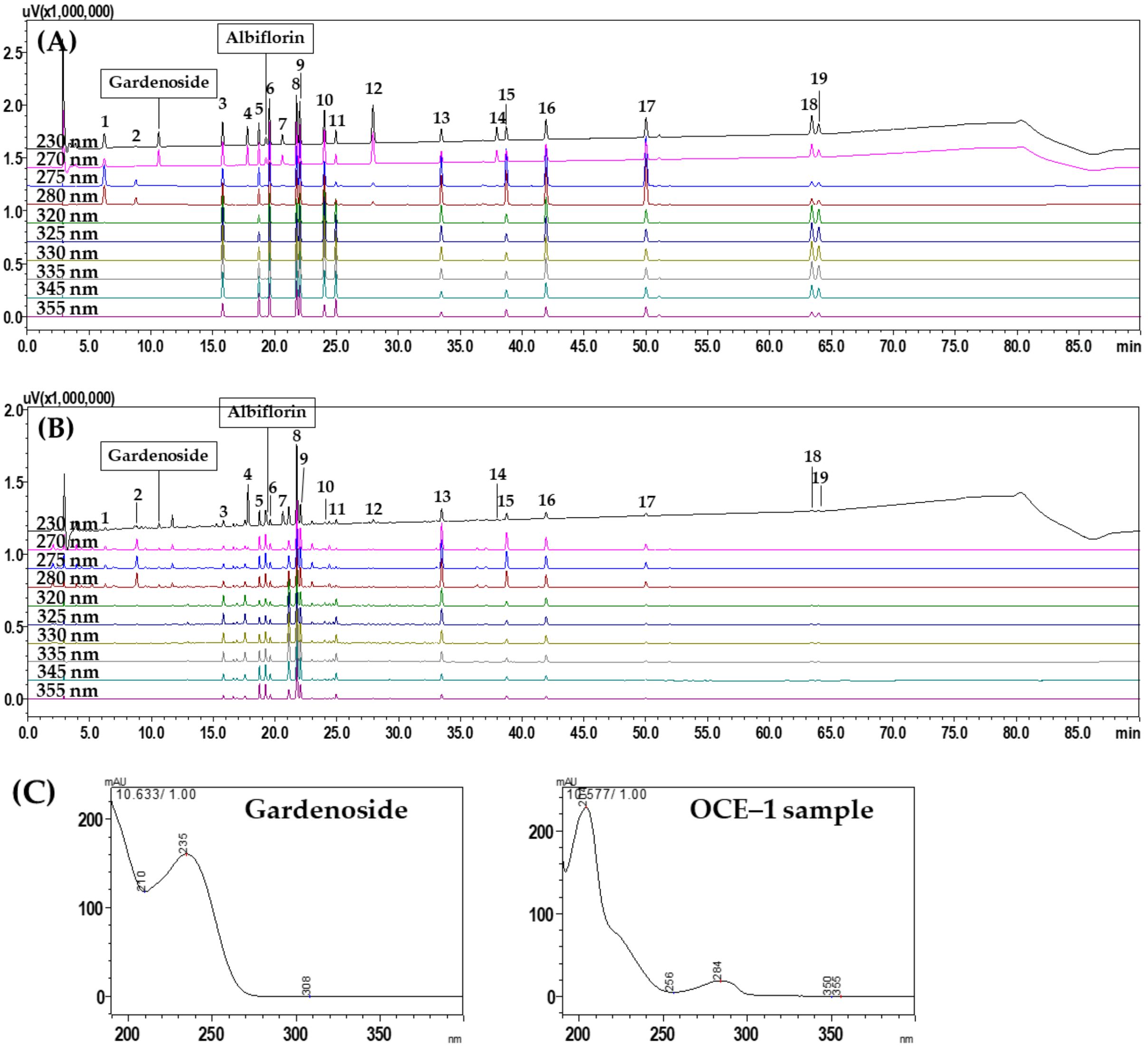

Figure S2. HPLC chromatograms of the solution of standard mixture (A) and 70% methanol solution of OCE–1 sample (B), and UV spectrum of gardenoside (C). Gallic acid (**1**), 5-(hydroxymethyl)furfural (**2**), chlorogenic acid (**3**), geniposide (**4**), coptisine chloride (**5**), jatrorrhizine chloride (**6**), paeoniflorin (**7**), berberine chloride (**8**), palmatine chloride (**9**), ferulic acid (**10**), nodakenin (**11**), benzoic acid (**12**), baicalin (**13**), benzoylpaeoniflorin (**14**), wogonoside (**15**), baicalein (**16**), wogonin (**17**), decursin (**18**), and decursinol angelate (**19**).

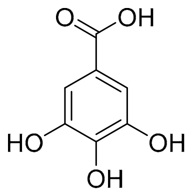

Gallic acid (**1**)

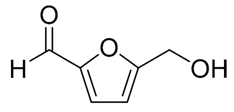

5-(Hydroxymethyl)furfural (**2**)

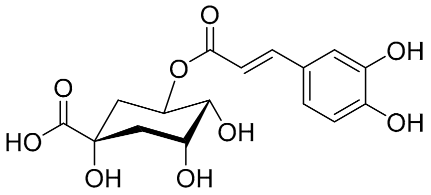

Chlorogenic acid (**3**)

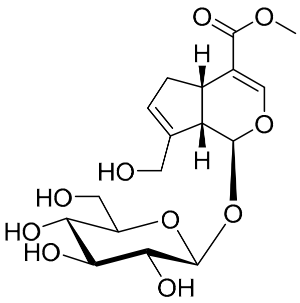

Geniposide (**4**)

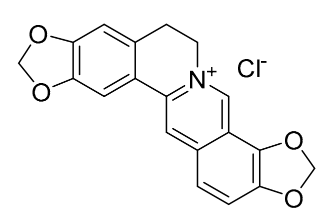

Coptisine chloride (**5**)

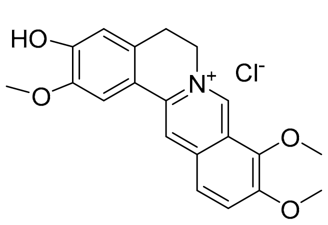

Jatrorrhizine chloride (**6**)

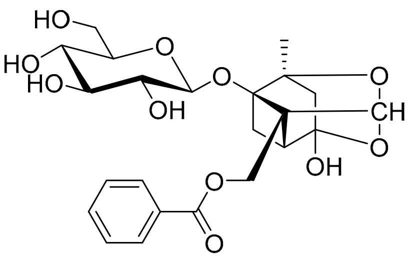

Paeoniflorin (**7**)

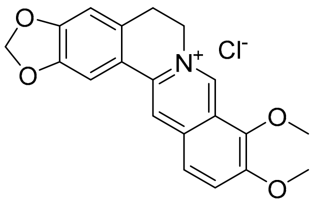

Berberine chloride (**8**)

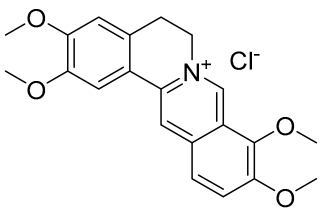

Palmatine chloride (**9**)

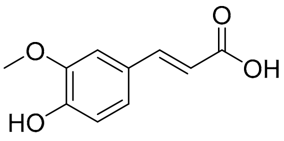

Ferulic acid (**10**)

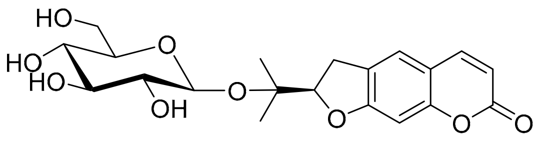

Nodakenin (**11**)

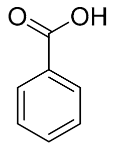

Benzoic acid (**12**)

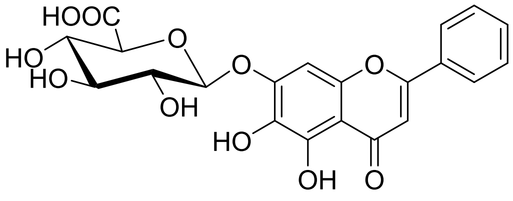

Baicalin (**13**)

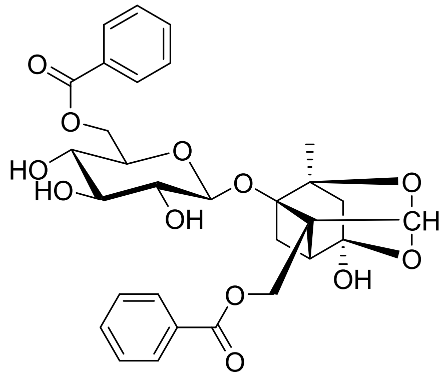

Benzoylpaeoniflorin (**14**)

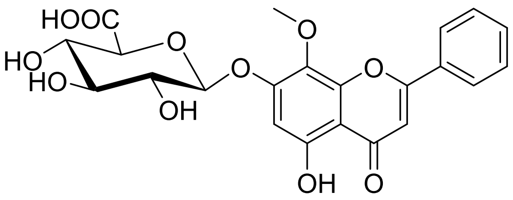

Wogonoside (**15**)

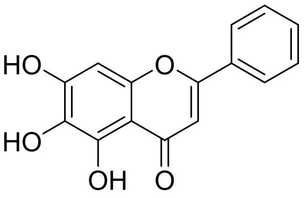

Baicalein (**16**)

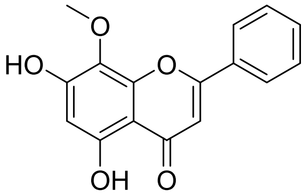

Wogonin (**17**)

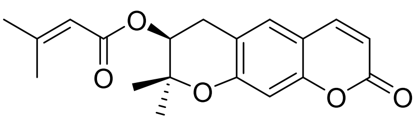

Decursin (**18**)

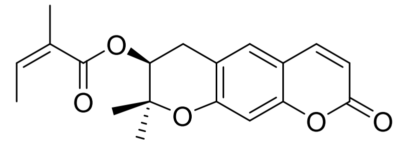

Decursinol angelate (**19**)

Figure S3. Chemical structures of the selected 19 marker compounds for quality control of OCE.

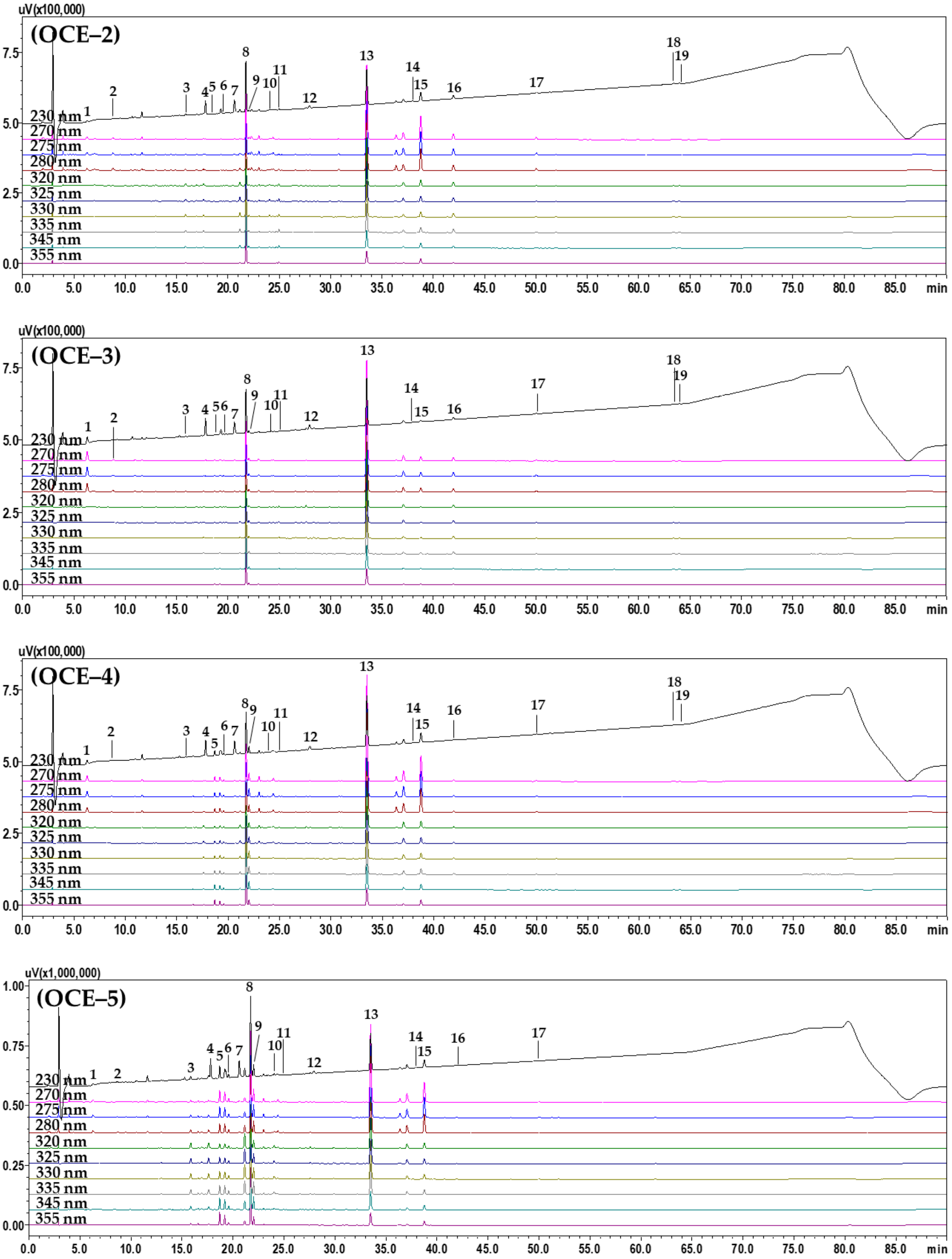

Figure S4. HPLC chromatograms of OCE–2 to OCE–5 samples. Gallic acid (**1**), 5-(hydroxymethyl)furfural (**2**), chlorogenic acid (**3**), geniposide (**4**), coptisine chloride (**5**), jatrorrhizine chloride (**6**), paeoniflorin (**7**), berberine chloride (**8**), palmatine chloride (**9**), ferulic acid (**10**), nodakenin (**11**), benzoic acid (**12**), baicalin (**13**), benzoylpaeoniflorin (**14**), wogonoside (**15**), baicalein (**16**), wogonin (**17**), decursin (**18**), and decursinol angelate (**19**).
